# Effects of age on a real-world *What-Where-When* memory task

**DOI:** 10.3389/fnagi.2015.00074

**Published:** 2015-05-18

**Authors:** Adèle Mazurek, Raja Meenakshi Bhoopathy, Jenny C. A. Read, Peter Gallagher, Tom V. Smulders

**Affiliations:** Institute of Neuroscience, Newcastle UniversityNewcastle upon Tyne, UK

**Keywords:** episodic memory, aging, *What-Where-When* memory, ecological validity, neuropsychology, dementia

## Abstract

Many cognitive abilities decline with aging, making it difficult to detect pathological changes against a background of natural changes in cognition. Most of the tests to assess cognitive decline are artificial tasks that have little resemblance to the problems faced by people in everyday life. This means both that people may have little practice doing such tasks (potentially contributing to the decline in performance) and that the tasks may not be good predictors of real-world cognitive problems. In this study, we test the performance of young people (18–25 years) and older people (60+-year-olds) on a novel, more ecologically valid test of episodic memory: the real-world *What-Where-When* (WWW) memory test. We also compare them on a battery of other cognitive tests, including working memory, psychomotor speed, executive function, and episodic memory. Older people show the expected age-related declines on the test battery. In the WWW memory task, older people were more likely to fail to remember any WWW combination than younger people were, although they did not significantly differ in their overall WWW score due to some older people performing as well as or better than most younger people. WWW memory performance was significantly predicted by other measures of episodic memory, such as the single-trial learning and long-term retention in the Rey Auditory Verbal Learning task and Combined Object Location Memory in the Object Relocation task. Self-reported memory complaints also predicted performance on the WWW task. These findings confirm that our real-world WWW memory task is a valid measure of episodic memory, with high ecological validity, which may be useful as a predictor of everyday memory abilities. The task will require a bit more development to improve its sensitivity to cognitive declines in aging and to potentially distinguish between mentally healthy older adults and those with early signs of cognitive pathologies.

## Introduction

Dementia is a degeneration of the brain and therefore of many cognitive processes, including memory. Memory deficits are often evident before any other signs of dementia are obvious (Masur et al., 1994; Bäckman et al., 2001; Jorm et al., 2005). Monitoring memory function can therefore be useful for early diagnosis of dementia, which in turn can help with the management of the disorder, potentially therapeutically slowing down the progression. For example, it has been shown that early deficits in episodic memory abilities can be indicative of the likelihood of a person developing Alzheimer's disease later on in life (Bäckman et al., 2001). Episodic memory is our memory for personally experienced episodes from our own past, which we typically experience as “Mental Time Travel”: a mentally re-experiencing of the episode in question (Suddendorf and Corballis, 1997).

One of the problems with using cognitive indicators as potential early-warning signals for dementia is that many cognitive capacities diminish as we get older. Processing speed, working memory, and long-term memory are all known to decline steadily as we age, although aspects of verbal short-term memory (e.g., digit span) and vocabulary may decline rapidly in later-life (Hedden and Gabrieli, 2004). With regard to long-term memory, while semantic processes are relatively unaffected, episodic memory exhibits a much greater degree of decline (Nyberg et al., 2012). Numerous studies have shown performance impairments in episodic-like memory tests in older people, even if there is no evidence of dementia or Mild Cognitive Impairment (MCI) (Harris et al., 2002). For example, Kessels et al. (2007) demonstrated broad performance decrements in older adults on a visuo-spatial episodic memory task which were especially pronounced in conditions requiring contextual binding. Tasks requiring the learning and recall of word lists (e.g., Rey-Auditory or California Verbal Learning Tests; R-AVLT/CVLT) have been found to be impaired in aging (Lundervold et al., 2014), with particular deficits in temporal order indices (Blachstein et al., 2012). There is also some suggestion that the age-related decline in verbal episodic memory may be greater in males than females (Lundervold et al., 2014). Because of these changes, it is sometimes difficult to distinguish the early signs of dementia from natural declines in cognitive capacity with old age. However, it has been suggested that measures such as the Rey-AVLT may be useful in delineating different dementias (Tierney et al., 1994; Ricci et al., 2012).

One potential criticism of many of the clinical tests of episodic memory is that they do not have very high ecological validity (Sbordone and Long, 1996). Everyday episodic memory typically has a number of characteristics that are not easily captured in most clinical tests: it is made up of long-term memories for unique events in their spatiotemporal context (what happened, where it was, when it was). The information is usually encoded in an incidental manner, and freely recalled, without any cues relating to the original event (Pause et al., 2013). Laboratory tests usually match some of these features, but rarely all of them. For example, some tests, like the R-AVLT, are about free recall of long-term (30-min) memory (in this case of a list of words), but the information is just a list of words (no spatiotemporal context needs to be remembered, although the optional temporal-order trial can be administered; Vakil and Blachstein, 1994); and it is learned in an intentional manner and rehearsed several times. Other tests (e.g., the Object Relocation task; Kessels et al., 1999) capture the binding between objects (what happened) and spatial locations (where it was); they typically do this over short retention intervals, using recognition processes for the items (though not for the locations) and again include intentional encoding of the information. The advantage of all these tests is that the experimenter/clinician knows exactly which answers are correct and which are wrong, because they control the information to be retained. When more ecologically valid measures of episodic memory are used, such as having people freely recall real events from their own lives, the scoring of these memories necessarily has to rely on the amount of detail recalled, rather than on the accuracy of these memories, as no objective record usually exists of the original event (e.g., Irish et al., 2011). In addition, episodes that are recalled are often ones that have been recounted many times in the past, and may therefore contain more semantic information than actual episodic recall (Pause et al., 2013). Despite these criticisms, existing tests of episodic memory clearly have been useful (e.g., Bäckman et al., 2001), but they may miss aspects of real-world episodic memory.

Recently, a number of new tests have been developed to try and overcome some of the drawbacks of the traditional tests and gain more ecological validity. Some of these tests are based on a reconceptualization of episodic memory which was originally adapted for use with non-human animals. In the absence of language, the tests are based on the animal experiencing two unique episodes, and then demonstrating through their behavior what is remembered about these two episodes. These tests emphasize the long-term retention of unique information about events in their spatiotemporal context. In the first study to do so, food-hoarding California scrub jays (*Aphelocoma californica*) hid two types of food on each of two separate occasions. Having been trained to know that the preferred food type degrades after several days, but the non-preferred one does not, they were then tested shortly after the second hiding episode. They only recovered the preferred food in the locations where they had hidden it in the second hiding episode, showing that they remembered which food (what) they had hidden in which locations (where) and on which occasion (when) (Clayton and Dickinson, 1998). Since then, several variations on this task have been developed for other animals, including other birds (Feeney et al., 2009; Zinkivskay et al., 2009; Gould et al., 2012), as well as rats and mice (Dere et al., 2005; Eacott et al., 2005; Babb and Crystal, 2006; Kart-Teke et al., 2006; Roberts et al., 2008).

More recently, adaptations of these tasks have been developed for humans. In a typical task, participants experience one or two unique events, and then have to recall what happened where, and when (Pause et al., 2010; Plancher et al., 2010; Hayne and Imuta, 2011; Holland and Smulders, 2011; Russell et al., 2011; Easton et al., 2012; Russell and Hanna, 2012; Cheke and Clayton, 2013; Inostroza et al., 2013; Saive et al., 2013, 2014; Newcombe et al., 2014; Weber et al., 2014). This is either in terms of “in which of the two episodes,” or “when in the episode,” asking about the sequence in which things happened. Some of these methods require an explicit response from the participants, but some try to assess memory purely based on behavioral responses (e.g., exploration behavior; Pause et al., 2010; Weber et al., 2014). All of these novel methodologies (with the exception of Holland and Smulders, 2011) use displays on a computer as the information to be remembered. However, we believe that this lacks the richness and complexity of real-world situations, which are part of natural episodic memories, and may therefore be less natural for older people to interact with. A real-world task may also have better real-world predictive value.

In the current study, we use a further adapted version of the task first reported by Holland and Smulders (2011). In this task, participants hide eight different objects in eight different locations (indicated by the experimenter) in a real-world room on each of two occasions on the same day (see Materials and Methods for details). After another 2 h, participants are then taken back into the room and asked to recall which objects they have hidden where, and on which occasion. The participants are told a cover story about the study, so that they would encode the information incidentally, rather than intentionally. Therefore, this task tests relatively long-term memory (>60 min; Pause et al., 2013) for incidentally-encoded information about unique and somewhat unusual (and hence arousing) episodes in their spatiotemporal contexts, hence fulfilling all seven of Pause et al.'s (2013) criteria for a good test of episodic-like memory. We also ask them about their subjective experience of the recall, fulfilling the criterion for real episodic memory as well. Part of the memory retrieval is based on free recall, although the spatial locations are in view of the participant and could therefore be solved using a familiarity mechanism. Because the participants move around a real environment and interact with real objects and locations, the task has added ecological validity over computer-based or paper-based tests. Because the objects are all unique, the task also allows us to test object memory and spatial memory independently of the memory for how different features of the episodes are bound together. The goal of the study was to investigate whether older participants would show a deficit in this novel test of episodic memory, and to compare their performance to other cognitive tasks in which age differences are well established.

## Materials and methods

### Participants

Fifty eight people participated in the study, which was approved by Newcastle University's Faculty of Medical Sciences Ethics Committee (approval number 00414), and run between January and May 2012. Because we did not know which effect size to expect, we decided on a sample size similar to that which had allowed us to detect performance differences in a previous study (Holland and Smulders, 2011). The sample was composed of two age groups: 26 young adults (17 women and 9 men, mean age 20, ranging from 18–24, all students), and 32 older people (19 women and 13 men, mean age 70, ranging from 61–85). One of the older participants had a visual impairment which prevented them from reading, so tasks that involved reading words or numbers were not administered to this participant. Each participant spoke English as a native language or spoke it fluently enough to study at a UK higher education establishment. All participants underwent the same procedure. At the end of the experiment, older people received a ₤ 20 gift card for a shopping center, students of the School of Psychology were given participation credit for their degree and other students were paid ₤ 5.

### Procedure

Participants attended the lab twice in the same day. In the morning session, they were briefed on the procedures and filled out consent forms. They then performed the first session of the *What-Where-When* (WWW) task, adapted from Holland and Smulders (2011). They then went away for approximately 2 h, during which they had lunch. After lunch, they first performed the second session of the WWW task. They were then run through a battery of other neuropsychological tasks, before being tested for their memory in the final WWW session. Details about the exact procedures for the different tasks can be found below.

#### *What-Where-When* task

**Figure 1:**
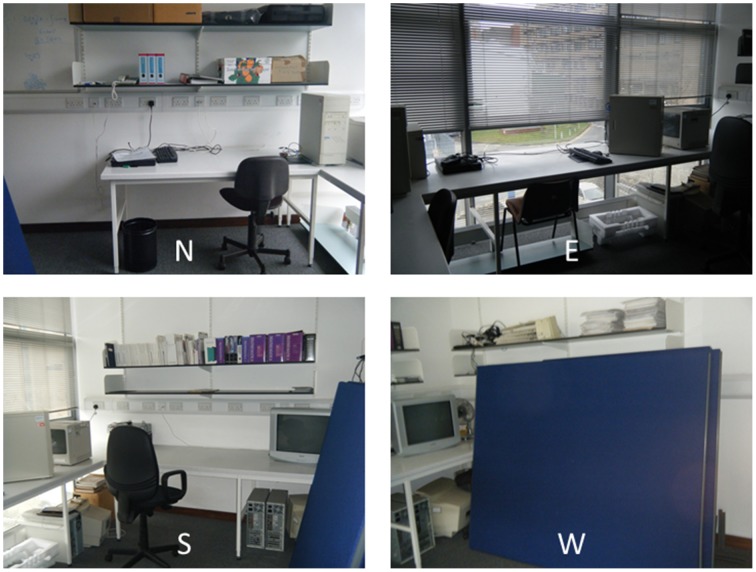
**A view of the four walls (N, North; E, East; S, South; W, West) of the room in which the WWW memory task was run**.

The WWW task was conducted with all participants unaware that they were participating in a memory task. They were told that the aim of the study was to investigate how well they could repeat a sentence (“She bought a bit of butter”) again and again under distracting conditions, and whether practice improved their performance. They were made to believe that their voice was being recorded. In addition to being part of the cover story, the sentence also served as articulatory suppression (Hanley, 1997), to prevent participants from verbally rehearsing any information during the task. In the first session, participants were required to hide eight objects (*an earring, a spoon, a coin, a pencil top, a toy frog, a party blower, a fold-back paperclip, and a playing card*) in pre-determined locations in a cluttered office room (Figure 1). The objects were given to the participant one at a time, and the locations were identified during the task by the experimenter pointing at the locations for the participant to place an object in. The locations were the same for every participant, and had been chosen to be distributed throughout the room, so that the temporal information was not confounded with the spatial information.

The second session occurred in the afternoon, on average 2 h after the first session. First, participants were required to perform the same procedure as in the morning session with eight other objects (*a key, a plastic ball, a clothes peg, a rubber band, a bottle cap, an eraser, a top, and a toy snake*) in eight new pre-determined locations. Finally, after having been tested on all the other neuropsychological tests (see below), the participants were returned to the room in which they had hidden the objects, and asked to recall which objects they had hidden in which locations and on which of the two occasions. They were also encouraged to report any incomplete information they could recall (e.g., items for which they could not remember the locations or vice versa). After they had recalled all the information they could, they were asked how they experienced the recall of the information: whether they re-experienced the hiding events in their heads (“remember”), or whether they just knew the information (“know”). They were also asked how vividly they re-experienced the information on a scale from 1 to 5, based on the Vividness of Visual Imagery Questionnaire (Marks, 1973).

#### Memory self-assessment

Right after the second hiding session, participants filled in three self-evaluation questionnaires: the Memory Complaint Questionnaire (MAC-Q) (Crook et al., 1992) and Every Day Memory Questionnaire (Sunderland et al., 1983) were used to assess perceived memory problems and the Geriatric-Depression-Scale questionnaire (GDS) was used to assess the general mood of the participants (Greenberg and Kurlowicz, 2007).

Then, a battery of neuropsychological tests was performed. The exact order was designed such that shorter tests could be run during the retention intervals of the longer tests. The total duration of the test battery was 2 h, including short breaks. We present the tests here in order of their complexity.

#### Rey auditory verbal learning test (R-AVLT) (Rey, 1964)

Participants listened to a list of 15 words (1 s between presentations; List A), which had been recorded using Audacity 1.3 beta by a native English speaker. They were then asked to immediately recall this list (measure A1). After this, a learning phase was carried out during which participants were presented with the list four more times and after each presentation they were again asked to verbally recall the list (A2–A5). Immediately after the fifth recall, participants were required to memorize a new list of 15 words (List B) and asked to immediately verbally recall them (B). Then without further presentation, participants were asked to think back and recall as many words as possible from List A (A6). The output of this test included a measure of retroactive interference (RI = A5–A6) and proactive interference (PI = A1–B) scores. Then, around 30 min later, without further presentation, participants were again required to recall the words from list A (A7).

Following the delayed word recall (A7) there was a word recognition task of the 30 words from List A and List B. The participants were presented with 50 words (the 30 words from lists A and B, plus 20 new words), and taken through this list by the experimenter. For each word, they needed to identify whether it was a new word or not, and if not, which list it belonged in. Temporal order judgment assessment followed the recognition trial: participants had to reorganize 15 pieces of paper on which the words of list A had been written in the correct order. The same procedure was used for the words of list B. We used three different measures of how well the reconstructed order matched the original order: (1) Hits: the number of words correctly placed at their original serial position; (2) Absolute deviation: this score was calculated by summing the absolute deviation of each word from its original position (range of scores: 0–14); (3) Correlation: Pearson product-moment correlation calculated for each subject, between the listed order and the true order (Vakil and Blachstein, 1994).

#### Object relocation (Kessels et al., 1999)

This paradigm is made up of five different test conditions: an Object Recognition Memory (ORM), in which participants have to memorize and then pick out 10 objects (from a choice of 20); a Visual Spatial Reconstruction (VSR), in which a spatial array of identical objects is shown on one side of a computer screen, and the participants have to copy it on the other side of the screen; a Position Only Memory (POM), in which 10 identical objects are presented on the screen for the participant to memorize, and then reconstruct after a retention interval; an Object Location Binding (OLB), in which 10 different objects are presented on the screen to be memorized, which then need to be matched to indicated locations after a retention interval; and the Combined Object Memory (COM), which is a combination of POM and OLB, in that 10 objects and locations need to be memorized, and the locations are not shown after the retention interval. For every condition, there was first a practice trial with fewer objects/locations, followed by two full trials with 10 items each. For the memory versions of the task (ORM, POM, OLB, and COM), we had one trial with a zero-second retention interval, and one with a 3-min retention interval. Half the participants did the short retention interval first, and half did the long retention interval first. The outcome measures for the ORM and OLB are the number of correctly identified objects/locations, whereas for the other three tasks, the outcome measure is the sum of the absolute distances between the objects and their correct locations (or in the case of the POM, the nearest correct location).

#### Standard neuropsychological tests

Verbal working memory was tested using the Forward Digit Span, while verbal working memory combined with executive function was tested using the Backward Digit Span (Wechsler, 1981; Lezak et al., 2004). We used the maximum span remembered as the outcome measure for both tests. Visual working memory was tested using the CANTAB (Cambridge Cognition, Cambridge, United Kingdom) version of the Corsi Block task (Spatial Span–SS), the CANTAB Paired Associates test and the Visual Patterns Test (Della Sala et al., 1997). Psychomotor speed was tested using the Trail making Test A, and psychomotor speed plus executive function using the Trail making Test B (Lezak et al., 2004). Finally, language comprehension was tested using two subtests from the Speed and Capacity of Language Processing (SCOLP) test: the SCOLP Word and the SCOLP Comprehension tests (Baddeley et al., 1992).

### Data analysis

#### Classic statistics

All data analyses (except for the Bayes Factor calculations, see below) were performed in IBM® SPSS® v21. For normally distributed interval data, we used a General Linear Model (GLM) approach, which gives classic *F*-values as the output. For counts of correct responses (e.g., SCOLP, AVLT, WWW), we used the Generalized Linear Model (GzLM) approach, with data from a binomial distribution with logit link function; for repeated measures of the same, we use the Generalized Estimating Equations (GEE), with the same link function, and an unstructured correlation matrix. The GzLM and GEE give Wald's χ^2^ as the output statistic. All models were simplified by removing non-significant interactions, starting with the highest-level interactions. For CANTAB errors, we used GzLM with data from a Poisson distribution with log-link function and for the Vividness scale, the data were treated as ordinal, using a log-link function. Results were considered significant at an α-level of 0.05. Descriptive statistics in the text and in the figures represent means ± SEM unless otherwise indicated.

#### Bayes factor

When differences between groups are not significant, this can be because of a “real” absence of a difference, or because of a lack of statistical power to detect a difference. One way to distinguish between these two options is to calculate a Bayes Factor (Dienes, 2011), which calculates how much more likely a given hypothesis is to be correct, given the data obtained. A Bayes Factor above one indicates that confidence in the hypothesis should increase, whereas a Bayes Factor below one suggests it should decrease. Online calculators exist to calculate Bayes Factors for comparisons of continuous variables between two groups (Dienes, 2011). However, the main dataset to which we wanted to apply the calculation was the outcomes of the WWW task, which consists of binary data (correct or not for each item, location or combination). We therefore designed our own Bayes Factor calculator for binary data. Details of this calculator can be found in the Appendix. Matlab code and an executable of the calculator itself can be downloaded from http://www.jennyreadresearch.com/research/matlab-code/bayes-factors-for-binomial-data/.

## Results

### Memory self-assessment

**Figure 2:**
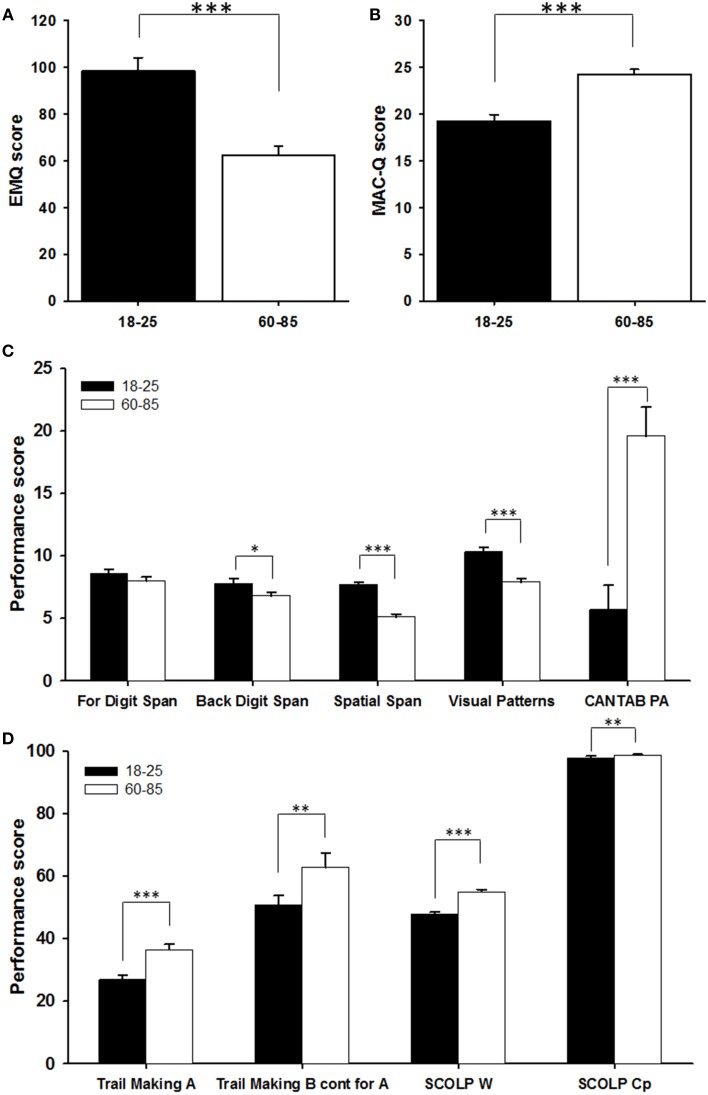
**Comparison of the two age groups on (A) the Everyday Memory Questionnaire (EMQ); (B) the Memory Complaint Questionnaire (MAC-Q); (C) verbal (Forward Digit Span, Backward Digit Span) and visual (Spatial Span, Visual Patterns, CANTAB PA) working memory tasks; and (D) psychomotor speed (Trail Making A), Executive Function (Trail Making B) and vocabulary (SCOLP tasks: W, Words; Cp, Sentence Comprehension)**. We plotted means + SEM; ^*^p < 0.05, ^**^p < 0.01, ^***^p < 0.001.

Older people reported fewer memory problems on the Everyday Memory Questionnaire (EMQ) than younger people [*F*_(1, 55)_ = 31.1, *P* < 0.001; Figure 2A], while people who reported more everyday memory problems also reported a lower mood on the Geriatric Depression Scale (GDS) [covariate in the GLM model; *F*_(1, 55)_ = 5.77, *P* = 0.020]. There were no age differences in scores on the GDS [*F*_(1, 56)_ = 0.039, *p* = 0.843] and the effect of mood on EMQ did not differ between the two age groups, so the non-significant interaction between age and GDS was left out of the GLM model. In contrast, in the Mac-Q, elderly people describe their memory as being poorer now than high school or college, more so than young people [*F*_(1, 55)_ = 19.38, *P* < 0.001; Figure 2B], and there was no effect of current mood on this self-report of memory performance [covariate; *F*_(1, 55)_ = 0.02, *P* = 0.886]. Again, the non-significant interaction between age and GDS was left out of the model. According to the criteria of Crook et al. (1992), a Mac-Q score =25 is associated with memory decline. By this standard, more than half (17/32) of elderly people were affected by age associated memory decline, while none of the young people were so affected [χ^2^_1_ = 19.54, *P* < 0.001].

### Working memory, executive function and knowledge

Participants were tested using a battery of measures for which age differences were expected based on previous literature. This served to verify that the sample was similar to previous samples of younger and older people. Older people performed worse on the visuospatial working memory tests (Figure 2C): the Spatial Span test [*F*_(1, 56)_ = 89.99, *P* < 0.001], the Visual Patterns Test [*F*_(1, 56)_ = 32.08, *P* < 0.001] and the CANTAB Paired Associates Test [χ^2^_1_ = 186.12, *P* < 0.001]. Whereas the two groups do not show a significant difference in performance on the Forward Digit Span [a test of verbal working memory; *F*_(1, 56)_ = 1.63, *P* = 0.207], older people perform worse than younger people on the Backward Digit Span, a test of executive function [*F*_(1, 56)_ = 5.38, *P* = 0.024]. As expected, older people were slower on Trail Making A, a test of psychomotor speed [*F*_(1, 55)_ = 18.60, *P* < 0.001; Figure 2D]. Controlling for psychomotor speed by using the time taken to complete Trail Making A as a covariate in the analysis of Trail Making B, a test of executive function, again indicates that older people perform worse on executive function than younger people [*F*_(1, 54)_ = 8.67, *P* = 0.005].

In contrast to measures of speed, working memory and executive function, older people outperformed younger people on the SCOLP tests of vocabulary [χ^2^_1_ = 92.53, *P* < 0.001] and sentence comprehension [χ^2^_1_ = 9.14, *P* = 0.003]. There were no age differences in the time in which participants finished the sentence comprehension task [*F*_(1, 55)_ = 2.53, *P* = 0.118].

### Rey-AVLT

#### Word recall and recognition

**Figure 3:**
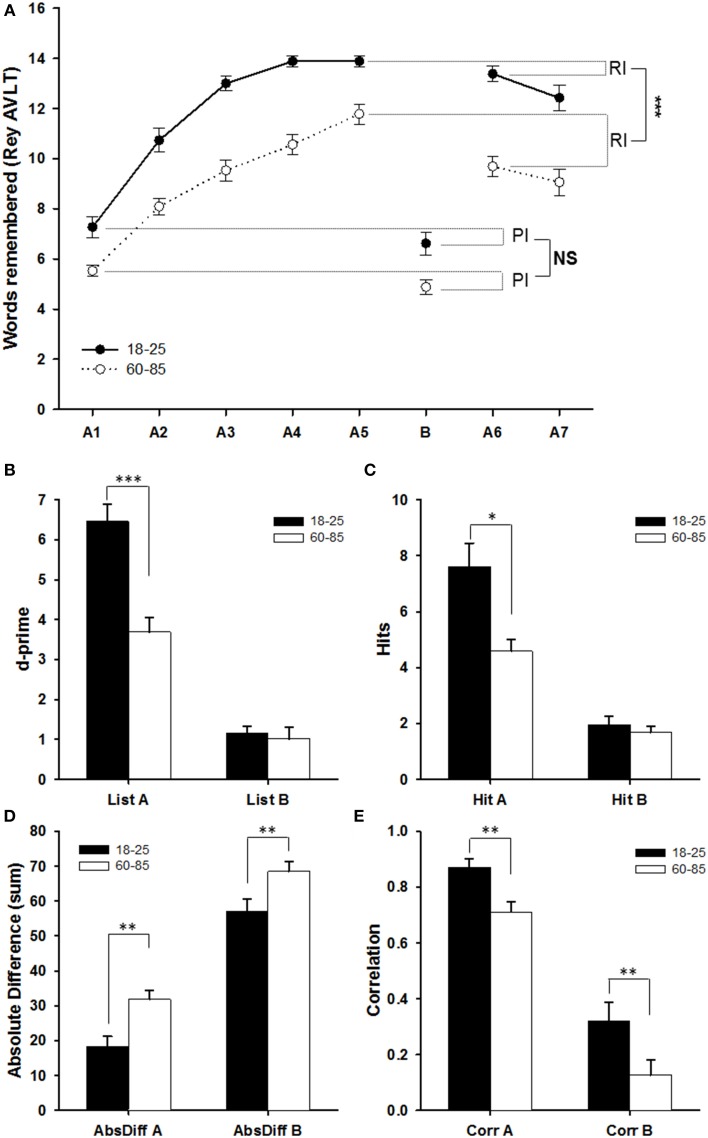
**Comparison of the two age groups in their performance on different measures of the Rey AVLT. (A)** Number of words (out of 15) recalled in the different phases of the task. RI, Retroactive Interference (A5–A6); PI, Proactive Interference (A1–B). **(B)** d-prime score on the recognition task. **(C–E)** Different measures of the memory for the order of the words in the list: **(C)** the number of words that were placed in their correct position (hits); **(D)** the sum of the absolute differences between the original position and the remembered position of each word in the list; **(E)** the Pearson's correlation coefficients between the original order and the remembered order. We plotted means ± SEM; ^*^p < 0.05, ^**^p < 0.01, ^***^p < 0.001.

In order to compare the learning and forgetting curves for the two age groups, a GEE analysis was performed with the different stages of the R-AVLT as within-subject factor and age as between-subject factor (Figure 3A). Older people remembered fewer words than younger people [χ^2^_1_ = 43.48, *P* < 0.001]. As expected, the number of words recalled increased from A1 to A5, and decreased from A5 to A7 [χ^2^_6_ = 416.26, *P* < 0.001]. The change over time was different for the age groups [interaction: χ^2^_6_ = 35.78, *P* < 0.001]. Looking at the learning curves from A1 to A5, older people consistently remembered fewer words than younger people [χ^2^_1_ = 39.66, *P* < 0.001], and both groups improved with repetition [χ^2^_4_ = 381.27, *P* < 0.001]. Again, the interaction between age and learning was significant [χ^2^_4_ = 31.55, *P* < 0.001], indicating that the change in performance was different between the older and the younger participants. Indeed younger participants did not significantly improve anymore from A4 to A5 (*post-hoc* pairwise comparisons, *P* = 1.00), whereas older participants continued to improve (Figure 3A).

The effect of the Retroactive Interference (of having list B between A5 and A6) was then examined. The age difference remained overall [χ^2^_1_ = 28.61, *P* < 0.001], and there was a significant retroactive interference effect [χ^2^_1_ = 34.81, *P* < 0.001], but the interaction between the two factors did not quite reach significance [χ^2^_1_ = 2.98, *P* = 0.084]. However, if the difference scores between A6 and A5 were examined using a GLM, the Retroactive Interference effect is much stronger in the older group [*F*_(1, 56)_ = 16.56, *P* < 0.001; Figure 3A]. Interestingly, although there clearly was an overall Proactive Interference effect of list A when retrieving list B [*F*_(1, 56)_ = 6.27, *P* = 0.015], there was no significant difference between young and old people in this effect [*F*_1, 56)_ = 0.003, *P* = 0.959].

Finally, the forgetting from A6 to A7 was investigated. Whereas younger people continued to outperform older people [χ^2^_1_ = 27.67, *P* < 0.001], and forgetting indeed occurred [χ^2^_1_ = 11.21, *P* = 0.001], this forgetting did not differ between the two age groups [χ^2^_1_ = 2.70, *P* = 0.10]. In this case, this lack of an age difference in forgetting was confirmed by the GLM comparing the difference scores between A6 and A7 [*F*_(1, 56)_ = 0.408, *P* = 0.526].

Participants were also asked to recognize the words from list A and list B in a larger list with 20 foils. d-prime was calculated for both lists, based on the number of hits (correctly recognized words) and false alarms (words attributed to the list that were not part of the list; Figure 3B). Performance was much better for list A than for list B [*F*_(1, 56)_ = 208.67, *P* < 0.001] for both age groups. Younger participants outperformed older participants [*F*_(1, 56)_ = 13.28, *P* = 0.001], but only for list A [interaction: *F*_(1, 56)_ = 20.71, *P* < 0.001], although this may be due to a floor effect for performance on list B.

#### Word order

The temporal order in which things happen is often cited as a crucial component of episodic memory. We had three measures of temporal order in recalling the word lists in the Rey-AVLT: Hit score (number of items in the correct position; Figure 3C), absolute deviation from correct position for each item (Figure 3D) and correlation between the real position and the recalled position (Figure 3E). We conducted either a GEE (hits) or an RM ANOVA (absolute deviation and correlation coefficients) with scores on list A vs. list B as the within-subjects factor and age as the between-subjects factor. Older people performed worse than younger people [lower hit scores: χ^2^_1_ = 5.68, *P* = 0.017; higher absolute deviation: *F*_(1, 55)_ = 12.15, *P* = 0.001; lower Pearson correlation: *F*_(1, 55)_ = 10.31, *P* = 0.002]. For both groups, performance was better for list A than for list B [higher hit score: χ^2^_1_ = 137.70, *P* < 0.001; lower absolute deviation: *F*_(1, 55)_ = 305.42, *P* < 0.001; higher Pearson correlation: *F*_(1, 55)_ = 191.34, *P* < 0.001]. There was a significant interaction between age and list for Hit score [χ^2^_1_ = 6.75, *P* = 0.009], but not for absolute deviation or correlation [Absolute deviation: *F*_(1, 55)_ = 0.22, *P* = 0.639; correlation: *F*_(1, 55)_ = 0.15, *P* = 0.699]. For the hits, it is possible that the age difference only exists for list A, not for list B. However, we should be cautious with this interpretation, as this is likely to be a floor effect for list B (<2 hits for all groups).

### Object location task

**Figure 4:**
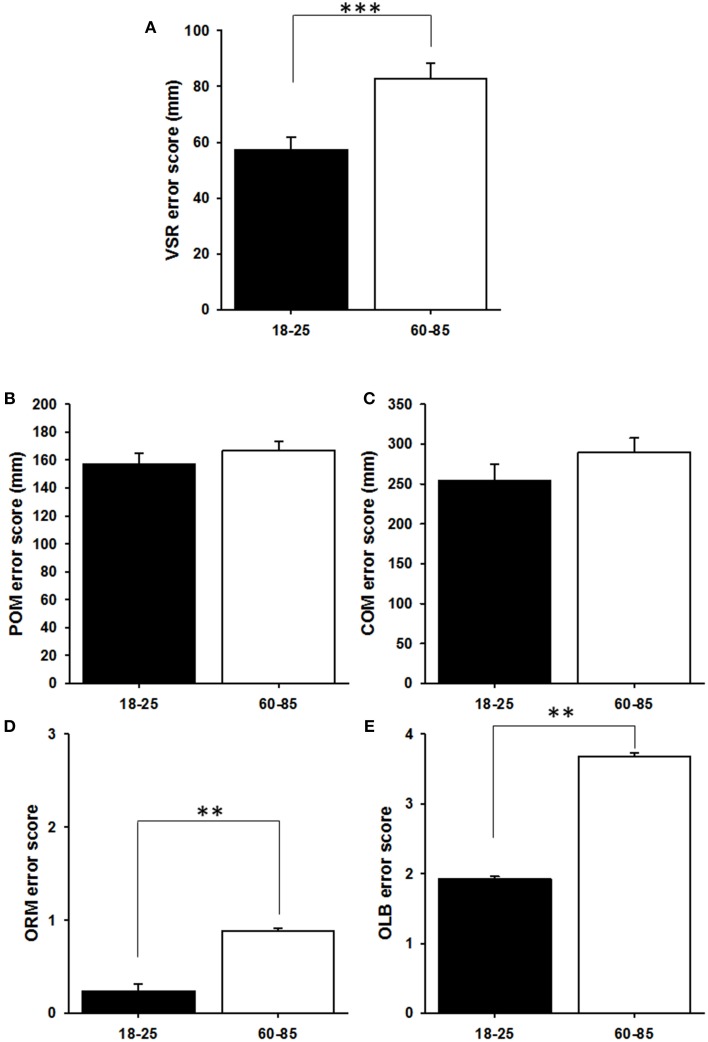
**Comparison of the two age groups in their performance on the Object-Location Binding task. (A)** Visuospatial reconstruction. The error score is the sum of the distance (in mm) between the original and reconstructed locations of the objects. **(B)** Position Only Memory. The error score is the sum of the distance between the remembered locations and the closest original locations of the objects. This score is statistically controlled for the error score on the VSR (see Materials and Methods). **(C)** Combined Memory Score. The error score is the sum of the distance (in mm) between the original and remembered locations of the objects. This score is statistically controlled for the error score on the VSR (see Material and Methods). **(D)** Object Recognition Memory. The error score is the number of incorrectly identified objects (out of 10). **(E)** Object-Location Binding. The error score is the number of marked locations with an incorrect object assigned to them (out of 10). We plotted means + SEM; ^**^p < 0.01, ^***^p < 0.001.

In the VSR task, younger people performed better than older people [*F*_(1, 55)_ = 13.13, *P* = 0.001; Figure 4A]. Because of this age difference in visuo-spatial perception, performance on VSR was controlled for when investigating age differences in spatial memory (POM and COM), by using the average VSR score across the two sessions as a covariate in the analysis. Thus controlling for worse spatial perception, no age differences were found in either Place Only Memory [*F*_(1, 54)_ = 1.35, *P* = 0.250; Figure 4B] or COM [*F*_(1, 53)_ = 2.04, *P* = 0.159; Figure 4C]. There was also no difference between the two delay conditions in either measure [POM: *F*_(1, 54)_ = 0.99, *P* = 0.325; COM: *F*_(1, 53)_ = 0.11, *P* = 0.739]. Age differences were found in the ORM task [χ^2^_1_ = 7.66, *P* = 0.006; Figure 4D] and the OLB task [χ^2^_1_ = 8.17, *P* = 0.004; Figure 4E]. Again, delay did not significantly affect performance on either of these two measures [ORM: χ^2^_1_ = 0.005, *P* = 0.946; OLB: χ^2^_1_ = 1.79, *P* = 0.181].

### Performance on the WWW task

#### Overall performance

**Figure 5:**
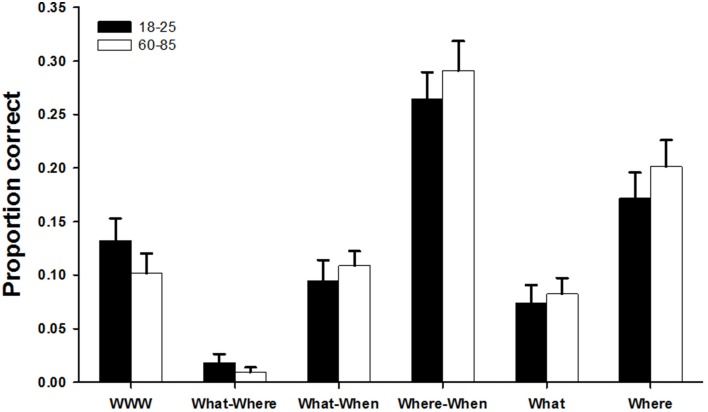
**Comparison of the two age groups on the WWW memory task**. The graph represents the proportion of correct objects in each of the categories, excluding all other categories (see Material and Methods). For example, proportion of correct *What-Where* combinations is out of the total number of objects that have not been remembered in a complete WWW combination, and the proportion of correct locations (*Where*) is out of the number of locations that have not been remembered in any combination at all. None of the differences are significant. We plotted means + SEM.

Performance on the integrated WWW measure did not differ between the two age groups [χ^2^_1_ = 2.10, *P* = 0.147]: young people remembered on average 2.12 ± 0.27 WWW combinations, while older people remembered 1.60 ± 0.22 correct combinations (Figure 5). This result does not allow us to conclude anything about age differences in this measure, as there is roughly the same probability that the groups do not differ from each other, as that the younger people outperform the older people (Bayes Factor = 0.93; for Bayes Factor calculation, see Methods and Appendix; Jeffreys, 1961; Dienes, 2011). Older people were more likely to not remember any complete WWW combinations (*n* = 11/32 participants) than younger people [*n* = 3/26; χ^2^_1_ = 4.09, *P* = 0.043]. Interestingly, when we use the actual age of the participants as a co-variate, within both the young and old groups, older people (in the range of our samples, so 24-year-olds in the younger group, and 70+-year-olds in the older group) performed better than younger people [main effect of actual age: χ^2^_1_ = 12.06, *P* = 0.001], and the slopes are different for the two groups [interaction between group and actual ages: χ^2^_1_ = 5.17, *P* = 0.023], which was most likely due to the fact that the range of performance was the same for both groups (0–6 combinations), but the age range was wider for the older participants.

Memory for incomplete combinations of *What-Where*, *What-When* and *Where-When* (not including the correct WWW combinations; Figure 5) was then examined. There were no significant age group differences in the performance on these combinations [χ^2^_1_ = 0.043, *P* = 0.835]. The performance on the different combinations was very different, however [χ^2^_2_ = 182.74, *P* < 0.001]. Few participants recalled any incomplete *What-Where* combinations (*n* = 50 did not recall any, *n* = 7 recalled 1 and *n* = 1 recalled 2), implying that when people recalled where a particular object was hidden, they also remembered on which occasion that had happened. Participants recalled more incomplete *What-When* combinations (on average 10 ± 1.2% of the combinations they had not recalled as a full WWW combination), and even more incomplete *Where-When* combination (on average 28 ± 1.9% of the combinations not recalled as full WWW combinations). This strongly suggests that it is possible and even likely to bind objects or locations to time frames by themselves, but when both object and location are recalled, the time frame is recalled as well. This pattern of performance across the three types of incomplete combinations did not differ significantly between age categories [interaction: χ^2^_2_ = 0.78, *P* = 0.677; Figure 5]. When we look at whether people recalled any incomplete combinations at all, there was no age difference in the number of people recalling at least one *What-Where* combination [χ^2^_1_ = 0.50, *P* = 0.481] and at least one *Where-When* combination (all participants recalled at least one of these). Older people, however, were more likely to recall at least one *What-When* combination than younger people [χ^2^_1_ = 3.90, *P* = 0.048].

Finally, performance on remembering individual objects or locations that had not been recalled as part of a combination of any kind was investigated. Similar to the incomplete combinations with *When*, locations were remembered much more commonly than objects [20 ± 1.8% of the locations not recalled in combination vs. 8 ± 1.1% of the objects not recalled in combination; χ^2^_1_ = 36.66, *P* < 0.001]. There were no differences between the age categories [χ^2^_1_ = 0.28, *P* = 0.594], nor was there an interaction between age and information retained [χ^2^_1_ = 0.048, *P* = 0.826; Figure 4]. Older and younger people were equally likely to remember at least one object [χ^2^_1_ = 0.38, *P* = 0.536] and at least one location [χ^2^_1_ = 0.10, *P* = 0.751].

The lack of significant age differences in the incomplete combinations and individual items could be due to a genuine absence of age differences, or due to lack of statistical power. In order to determine whether there really is no age difference, Bayes Factors were calculated for each of these five comparisons between young people and older people. In this study, the Bayes Factors for all these comparisons indicated that it was 4.5 to 15.5 times more likely that there really are no age differences than that the younger people perform better than the older people, suggesting the lack of difference is not due to a lack of statistical power. One exception is the comparison of the incomplete *What-Where* combinations, where no conclusion could be drawn due to the small number of responses in that category.

#### Subjective experience of WWW recall

**Figure 6:**
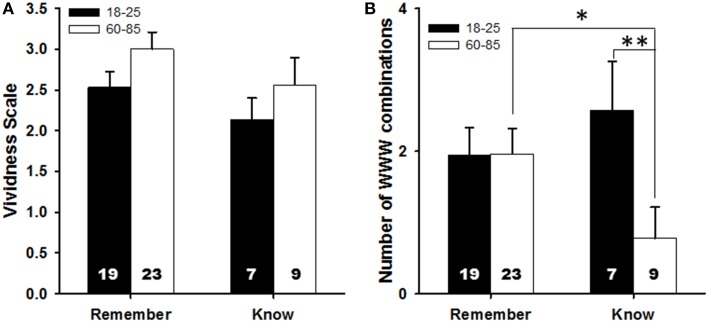
**Comparison of the two age groups on: (A) the average vividness score, split by those participants who claimed to re-experience the event (Remember) and those who just knew the information (Know)**. The numbers on the bars represent the number of individuals in each condition. **(B)** The mean number of WWW combinations recalled by participants, split in the same manner as in **(A)**. We plotted means + SEM; ^*^p < 0.05, ^**^p < 0.01.

In both age groups, participants claimed to “relive the session in their head” (“remember”) significantly more often than to just know (“know”) which objects were hidden where and when [χ^2^_1_ = 10.38, *P* = 0.001; in total *n* = 42/58], and this did not differ between the age groups [χ^2^_1_ = 0.01, *P* = 0.919]. People who claim to relive the sessions did not score their recall experience higher on the vividness scale [χ^2^_1_ = 1.88, *P* = 0.170]. There were no age group differences on the vividness scale [χ^2^_1_ = 2.63, *P* = 0.105], nor was there a significant interaction between age group and whether they remembered or knew [χ^2^_1_ = 0.025, *P* = 0.876; Figure 6A].

Whether the mode of recall affected accuracy in the recall of the full WWW combinations was then investigated (including age category and the interaction between mode of recall and age also in the model). Mode of recall significantly interacted with age category to affect recall of the full WWW combinations [χ^2^_1_ = 6.39, *P* = 0.011; Figure 6B]: younger people who claim to “just know” outperform older people who claim to “just know” [remembering 2.88 ± 0.61 vs. 0.80 ± 0.29 combinations, respectively; χ^2^_1_ = 6.72, *P* = 0.010]; but there is no age difference among those who claim to “remember” [young: 1.92 ± 0.30, old: 1.76 ± 0.27; χ^2^_1_ = 0.38, *P* = 0.536]. Looking at it in a different way, older people who “remembered” the episode performed better than older people who “just knew” the information [χ^2^_1_ = 5.73, *P* = 0.017], while no such difference was present for the younger people [χ^2^_1_ = 1.08, *P* = 0.299]. Increasing vividness of experience did not significantly improve memory outcomes [χ^2^_1_ = 3.57, *P* = 0.059].

Another way to approach the mode of recall is to investigate the order in which the information is recalled. A retrieval order that follows the order of the original experience might indicate a mental time travel strategy. The correlation between the order of recall of hiding locations and the order of hiding in those locations was therefore examined. This correlation did not differ between the age categories [*F*_(1, 55)_ = 1.31, *P* = 0.258], nor did it differ from zero across all participants [Intercept: *F*_(1, 55)_ = 0.24, *P* = 0.878], suggesting people are not following their original route mentally when recalling the information. The average number of ranks (absolute difference) that any given recalled location was from its original rank also did not differ among the age categories [*F*_(1, 55)_ = 0.92, *P* = 0.342].

#### Rey-AVLT and WWW recall

**Figure 7:**
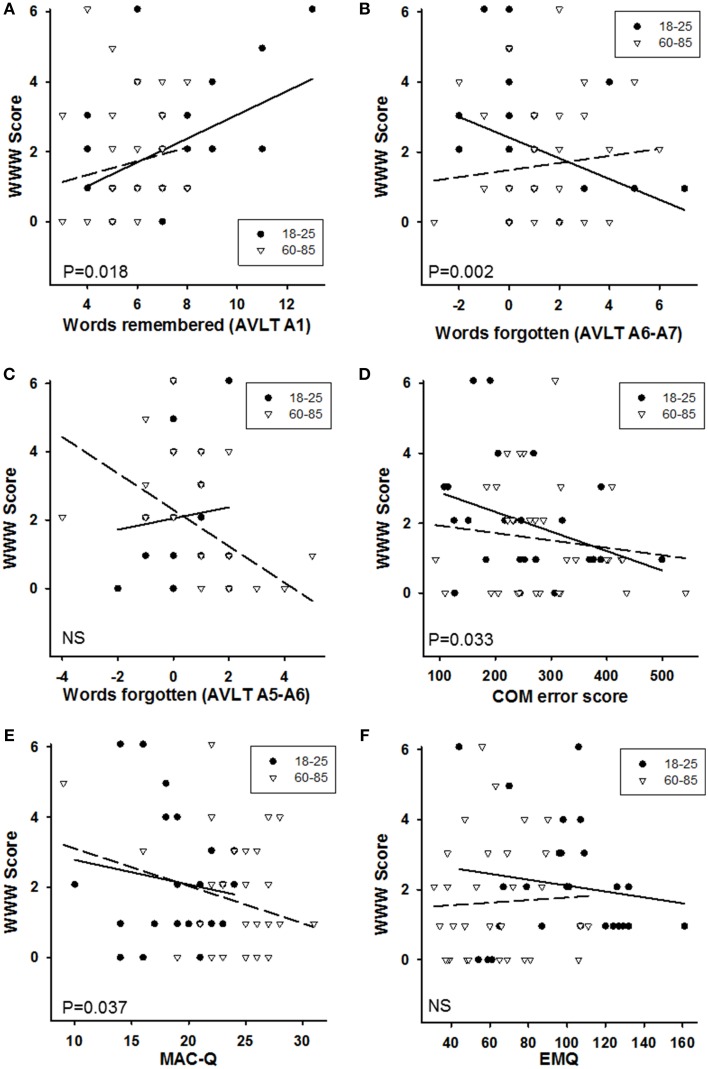
**Regression plots of performance on the WWW binding (number of correct combinations out of 16) as predicted by: (A) the number of words remembered after one reading of the list in the RAVLT (A1); (B) the number of words forgotten over the 30-min retention interval in the RAVLT (A6-A7; negative numbers indicate more correct words at A7 than at A6); (C) the number of words from list A forgotten while learning and repeating list B (A5-A6; Retroactive Interference; negative numbers indicate more correct words at A7 than at A6); (D) Error score on the Combined Object-Location Memory (note that one younger and one older participant had missing data for this task); (E) the Memory Complaint Questionnaire (MAC-Q; higher scores indicate more complaints); (F) the Everyday Memory Questionnaire (EMQ; higher scores indicate more problems)**. Continuous lines and filled circles: 18–25; long dashes and open triangles: 60–85. Significance levels indicated in the panels are for the overall effect of the predictor on the WWW performance. For more details of the analyses, see the main text.

Rey-AVLT and WWW are both purported measures of episodic memory. If this is the case, then individual variation in the each of the tasks should correlate across individuals. In order to test whether performance on the WWW combination was predicted by memory performance on the Rey-AVLT, another GzLM analysis was performed. Performance on a long-term memory task is dependent both on how much information was encoded in the first place, and how well this information is retained. For that reason, three measures from the Rey-AVLT were used to predict performance in the WWW task: the first was the total number of words recalled after a single exposure (A1), because in the WWW task, there was only one exposure to the information; the second was the number of words forgotten from A5 to A6 (A5-A6; Retroactive Interference, as the B list was learned between these two), and the final one was the number of words forgotten across the 30-min retention interval from A6 to A7 (A6-A7). The GzLM used these three variables as covariates and Age category as a fixed factor. Non-significant interactions between age and the three covariates were removed from the analysis in a stepwise manner until none remained. As reported above, there was no main age effect [χ^2^_1_ = 0.005, *P* = 0.942]. People who could memorize more words in one exposure also remembered more WWW combinations [χ^2^_1_ = 5.58, *P* = 0.018; Figure 7A], as did people who forgot fewer words from A6 to A7 [χ^2^_1_ = 9.96, *P* = 0.002; Figure 7B]. There was no significant effect of retroactive interference on remembering WWW combinations [A5-A6: χ^2^_1_ = 1.21, *P* = 0.271; Figure 7C].

#### Object location memory and WWW recall

Object Location Memory is another purported episodic memory task that should measure similar processes to the WWW task, and hence predict performance on the WWW task. Because there were no effects of memory delay on any of the outcome measures from the Object Location Memory task, mean performance across the two trials of each type for each participant was calculated. For the POM and COM measures, performance on the VSR was controlled for by calculating the residuals from a regression against VSR, and then adding mean performance across all participants to those residuals, in effect calculating the memory performance while keeping VSR performance constant. Using these four measures as covariates, only COM significantly predicted WWW memory performance [χ^2^_1_ = 4.56, *P* = 0.033], with individuals with more accurate object relocation performance being better in the WWW memory task (Figure 7D). There were no significant interactions with age.

#### Self-reported memory problems and WWW recall

Finally, the question of whether self-reported memory problems in the Mac-Q and EMQ predicted performance on the WWW task was explored. Using a similar analysis as above, people with a higher Mac-Q score (i.e., higher perceived memory problems) recalled fewer complete WWW combinations [χ^2^_1_ = 4.37, *P* = 0.037; Figure 7E], and this did not interact with age category. Interestingly, Mac-Q score did not predict performance on the COM task [*F*_(1, 53)_ = 0.88, *P* = 0.352], nor did it predict performance on the R-AVLT [χ^2^_1_ = 0.57, *P* = 0.451]. The scores on the Every Day Memory Questionnaire did not predict performance on the WWW test [χ^2^_1_ = 0.20, *P* = 0.657], nor were there any significant interactions (Figure 7F).

## Discussion

There are two main findings from this study. Firstly, the WWW memory task is a valid measure of episodic memory, as performance on the task is predicted by two other episodic memory tasks (RAVLT and Object Location Memory), independent of the age effects on these tasks. Secondly, younger people are more likely to remember at least one WWW combination than older people, and the age effect on this memory is mediated by how people recall the information.

### The WWW memory task measures episodic memory performance

Most participants reported using a “mental time travel” strategy (“remember”), rather than a semantic strategy (“know”) to recall the information in the WWW memory task. Using this mental time travel strategy significantly improved performance of the older people over not using it. Additionally, performance on the WWW combination memory task was predicted both by how many words participants could learn in one exposure to the word list (AVLT A1) and by how well they can retain the list over a 30-min retention interval. This suggests that the WWW memory integrates initial one-trial learning with long-term retention of information, key features of episodic memory (Pause et al., 2013). Performance on WWW memory was also predicted by the COM error score (controlled for visuospatial perception). This is not completely surprising, as the two tasks have very similar requirements: remembering the binding of objects to locations, and having a view of the potential locations at the time the memory recall is tested. Finally, the level of self-assessed age-dependent memory problems (MAC-Q) predicts performance on the WWW memory task. Interestingly, this was not the case for the EMQ. However, this instrument's value should be questioned in our study, because younger people reported more problems on this questionnaire than did older people (maybe because older people did not recall as many memory problems).

These findings therefore suggest that the WWW memory task draws on similar processes to other episodic memory tasks. The design of the task (remembering real objects, incidentally memorized in a real-world environment) additionally increases its ecological validity over existing tasks, making it potentially a better test of their practical memory skills. This is also indicated by the fact that the MAC-Q predicts performance on the WWW, but not on RAVLT or the COM score.

### WWW binding is affected by aging

Like in many other studies, we found that older people performed worse than younger people in a battery of cognitive tests, including visual and verbal working memory, executive function, psychomotor speed, and a classic episodic memory test (RAVLT). In contrast, they performed better on semantic knowledge tasks, such as vocabulary. All this is similar to what we already know about cognitive aging (Hedden and Gabrieli, 2004).

#### Object memory

In the WWW memory task, younger and older people remembered individual items and locations, as well as combinations of these with time, to a similar degree, as indicated by the Bayes Factors. In contrast to the lack of an age difference in remembering objects in the WWW task, there was a significant age difference in the ORM task, as had been found before (Kessels et al., 2007). However, in that task, the participants are presented with the objects and asked to indicate those they had seen before. In our WWW memory task, objects had to be freely recalled, which may make a difference, since Plancher et al. (2010) also failed to find an age difference for the free recall of objects along a virtual path. In addition, in our WWW study, very few objects were recalled at all (whether alone or in combination). This is probably because participants were given the objects in their hands and told to hide them in the indicated locations. Because they believed the objects to be a distractor, they may not have paid much attention to what was put into their hands. Object memory may be improved (and potentially made more sensitive to aging) by making people select the objects themselves, forcing them to pay attention to them.

#### Spatial memory

While not finding an age effect on object memory, Plancher et al. (2010) did find age differences in spatial memory performance in their virtual WWW task. We did not find an age difference in spatial memory in the WWW task. Plancher et al.'s (2010) spatial memory performance was assessed differently from ours, though. Whereas our participants were put back in the same environment, and could use spatial cues to trigger their memories, Plancher et al.'s (2010) participants were asked to describe where different features occurred along a virtual road through a virtual town, and to draw a map of this virtual road. Their memory testing was therefore completely free recall, whereas ours was not. We will test the difference between free recall and cued recall in a future study to ascertain the effect of the assessment method on performance and on the age-sensitivity of this performance. Interestingly, we also did not find an age effect in the Place Only Memory task, which is surprising, as Kessels et al. (2007) did detect an age effect on Place Only Memory using the same task. This may indicate that our sample may be less affected in spatial memory in general, and therefore our lack of age differences in the WWW memory task needs to be interpreted cautiously.

#### *What-Where(-When)* memory

Only when an object had to be linked to a location (which in almost all cases was also linked to the correct episode; see also Russell and Hanna, 2012), did we find an age deficit in the WWW memory task. There was no significant difference in the actual performance score, but significantly more older than younger people failed to recall a single WWW combination. Nevertheless, others performed as well or better than most young people, suggesting this performance may be spared in some mentally very healthy older people. We also found an age deficit in the Object-Location Binding task, in which participants are presented with a number of locations, and have to recall which object goes with which location. This is very similar (although not identical) to what is required in the WWW task. These findings agree with a range of other studies which have all detected age deficits in binding objects or odors to (spatio-temporal) context (Kessels et al., 2007; Plancher et al., 2010; Kinugawa et al., 2013). Interestingly, Plancher et al. (2010) found the binding deficit in the virtual environment only in their incidental encoding condition, but not in the intentional encoding condition.

One conclusion to draw from these findings is that our WWW memory test does not seem to be as sensitive to the effects of aging as some of the other published episodic memory tests. As mentioned before, the fact that relatively few objects were remembered at all severely limits the dynamic range of the possible responses, and therefore the potential for detecting more subtle differences between the age groups. In future versions of the task, we will correct this as described above. In addition, as pointed out earlier, being taken back to the actual spatial environment in which the information was encoded may help people by triggering more memories when seeing the locations again. It is possible that this helps older people more than it does younger people, again reducing the sensitivity of our task. In future versions, we will also change the test to completely free recall. We hope that these alterations will improve the sensitivity of our novel episodic memory task.

While the task can clearly be improved, the fact remains that we did see more older people failing to remember any combinations, while others outperformed many younger participants. Why might we see such a variable performance in our older sample? One thing that distinguishes our WWW test from all our other tasks is that it has much higher ecological validity. Whereas most neuropsychological tests on which older people show impairment require people to sit down with pen and paper or in front of a computer and effortfully memorize information or complete a task under time pressure, the WWW task is a real-world task, in which information was encoded incidentally, rather than intentionally. The ecological validity might make it easier for mentally healthy older people to apply more efficient or effective strategies that they have honed in everyday life (Hedden and Gabrieli, 2004). This would be less likely for more typical neuropsychological tests, including the RAVLT. This in turn suggests that our task might help us distinguish mentally healthy older people from older people with potential early signs of memory pathologies, such as dementia or MCI. We did not test this hypothesis in our study, as we did not attempt to diagnose early signs of dementia in our participants, but Plancher et al. (2012) found that healthy older people outperformed patients with amnestic MCI and with Alzheimer's Disease on a virtual WWW memory task. Plancher et al. (2010) also found that many older people struggled with the virtual version of the task, however, making our real-world, low-tech version potentially more user-friendly in the memory clinic.

One strange finding in our results was that in both younger and older participants, the people at the higher end of the age-range outperformed people at the lower end. This pattern does not fit any biological explanation, and we assume that it is due purely to the relatively small sample size in this study, resulting in very few people of any given age within each age group.

We did find that age effects on WWW memory were strongly influenced by the participants' subjective report of how they experienced retrieving the information. For the majority of people who experienced the memory retrieval as “remembering” (which we interpret as a mental time-travel or episodic strategy), the age difference was small. However, for the minority who experienced the memory retrieval as “knowing” (which we interpret as a semantic strategy), older participants performed worse than younger participants, and indeed than other older participants who “remembered” the information. Older participants were not more likely to use a semantic strategy than younger people, suggesting there is no age deficit in the ability to use an episodic strategy. Instead, it seems to suggest that older participants who either choose to or are forced to use a semantic strategy cannot do so as efficiently as younger participants. This finding deserves some further examination in the future. In a recent meta-analysis of recognition-memory tests (which is different from our partially free-recall procedure), aging was found to affect “knowing” less than “remembering,” which is the opposite of what we have found here. However, people with diagnosed Alzheimer's disease suffered equally on “remembering” and on “knowing” (Koen and Yonelinas, 2014). This again suggests that maybe some of our older people might have had undiagnosed mental pathologies and therefore showed a larger deficit when using a semantic strategy. This remains pure speculation, however.

### Conclusion

Memory for the binding of objects with locations (and occasions) in a long-term incidentally-encoded memory task was sensitive to aging, but the performance of older participants varied strongly, including some remembering as many or more combinations than most of the younger participants. This suggests the hypothesis that the WWW memory task could measure resilience to the normal cognitive declines of aging in mentally healthy individuals, yet be sensitive enough to pick up very early signs of age-related pathology. Only a larger cohort study with longitudinal follow-up to ascertain the development of such pathologies would allow us to test this hypothesis. Our test of WWW binding is simple to administer and does not require any special equipment (e.g., virtual reality suite or even a computer), making it more user friendly, especially with older people. The task will need a bit more development to make it more sensitive (larger dynamic range) and will need to be tested with identified patient populations, but we believe it shows promise as a simple and ecologically valid screening task for every-day episodic memory problems.

## Authors contribution

AM collected the data and did the first analyses and drafting of the manuscript. RB analyzed the order data of the WWW task. JCAR developed the software for the Bayes Factor analysis. PG and TVS developed the original concept and design of the study, trained AM and RB in the relevant tasks and analyses, and further analyzed the data. All authors contributed to the writing of the final manuscript and commented on drafts. All authors accept responsibility for the final manuscript and the results described therein.

### Conflict of interest statement

The authors declare that the research was conducted in the absence of any commercial or financial relationships that could be construed as a potential conflict of interest.

## Appendix: bayes factor calculation for binary data

To calculate Bayes Factors, our model assumes that the success probability (probability of getting a trial correct, for a given definition of correct in that particular analysis) is affected only by the grouping of interest, and is the same for all trials conducted by all subjects in a given group. This allows us to use simple binomial statistics. In the text below, we will speak about “young” and “older” groups, but the principle applies to any two groups being compared to each other.

We write N_Y_ for the total number of trials completed by all Young participants, and M_Y_ for the number of these which were successful; N_O_, M_O_ are analogous quantities for Older participants. The observed difference in the proportion of successful trials between these two age-groups is then

$$D=M_{\text{Y}}/N_{\text{Y}}-M_{\text{O}}/N_{\text{O}},$$

where a positive difference means that young people did better. We assume the mean success probability, averaged over both age-groups, is the observed proportion of successes when both groups are combined:

$$\mu =(M_{\text{Y}}+M_{\text{O}})/(N_{\text{Y}}+N_{\text{O}}).$$

By definition, the underlying (population) success probability for younger participants is then π_*Y*_ = μ + Δ/2, and that for older participants is π_O_ = μ – Δ/2. Clearly, both of these probabilities must lie in the range [0, 1]. Thus, the assumed mean success probability, μ, constrains the possible values of the true difference Δ. For example, consider the most extreme situation when the true probability is 0 for one group. In order to get a mean probability of μ for both groups, the true probability for the other group must be 2μ. It cannot go above this and still keep the mean probability at μ, since the probability for the first group cannot be negative. These considerations imply that Δ must lie between ±Δ_lim_, where Δ_lim_ = 2μ for 0 < μ ≤ 0.5 and Δ_lim_ = 2(1–μ) for 0.5 < μ ≤ 1.

We now want to compute the likelihood of the observed (sample) difference in the proportion of successful trials, D, if the underlying (population) difference in success probability is really Δ. We write this as Pr(D|Δ). To calculate this, we consider all the possible scores which would give the observed value of D, given the number of trials actually performed by each age-group. The probability of each score is given by the simple binomial distribution:

$$\mathrm{Pr}\left(m|N,\pi \right)=\frac{N!}{m!\left(N-m\right)!}\pi^{m}{\left(1-\pi \right)}^{N-m}$$

We sum the product Pr(m_Y_|N_Y_, π_Y_)Pr(m_O_|N_O_, π_O_) over all pairs of (m_Y_, m_O_) which satisfy 0≤m_Y_≤N_Y_, 0≤m_O_≤N_O_ and (m_Y_/N_Y_ − m_O_/N_O_) = D. This is our estimate of Pr(D|Δ): the probability of observing a particular difference D in the proportion of successful trials, given an actual difference Δ in the probability of a successful trial.

To compare the null hypothesis that there is no difference in success probability between age-groups, Δ = 0, with the experimental hypothesis that Δ could be non-zero, we computed the Bayes Factor, B. This is the ratio of the likelihood of the observed difference D under the experimental hypothesis to its likelihood under the null hypothesis, B = L_expt_/L_null_. These are

$$L_{\text{null}}=\text{Pr}\left(D|\Delta =0\right);\;L_{\text{expt}}=\int_{-\Delta_{\text{lim}}}^{+\Delta_{\text{lim}}}d\Delta \text{P}(\Delta )\text{Pr}\left(D|\Delta \right),$$

where Pr(D|Δ) is calculated as described above and P(Δ) is the *a priori* distribution for Δ under the experimental hypothesis. In our analysis, we set this distribution as a half-gaussian in the direction of younger people doing better:

$$\text{P}(\Delta )=\frac{1}{2\sigma \sqrt{2\pi }}\text{exp}\left(\text{​}-\frac{\Delta^{2}}{2\sigma^{2}}\text{​}\right)\text{for }\Delta \geq 0\text{ and P}(\Delta )=0\text{ otherwise}.$$

The standard deviation was set to half of the maximum possible difference in success probability between the two groups, σ = Δ_lim_/2. This sets the maximum difference at the 95% confidence interval, as suggested by Dienes (2011). From the expressions above, this means that σ = μ or (1–μ), whichever is smaller. In our data-set, μ < 0.5, so the standard deviation σ of our prior distribution for the difference in probability between the groups is equal to the estimated mean probability across both groups.

MATLAB code for this analysis is available at http://www.jennyreadresearch.com/research/matlab-code/bayes-factors-for-binomial-data/.
